# Intravenous anesthesia with high-flow nasal cannula improves recovery in elderly undergoing ureteroscopic lithotripsy: a prospective controlled study

**DOI:** 10.1186/s12871-024-02869-z

**Published:** 2025-01-02

**Authors:** Lifei Tang, Ran Guo, Yaochen Quan, Haiwen Zhang, Yingcong Qian, Youjia Yu, Shaoyong Song, Jian Li

**Affiliations:** 1 Department of Anesthesiology, The Fourth Affiliated Hospital of Soochow University, Suzhou, Jiangsu 215124 China; 2 Department of Anesthesiology, The 904 th Hospital of the Joint Service Support Force of the Chinese People’s Liberation Army, Wuxi, Jiangsu 214000 China; 3 Department of Pain Medicine, Zhejiang Provincial People’s Hospital, Hangzhou, Zhejiang 310014 China; 4 Department of Anesthesiology, Suzhou Xiangcheng People’s Hospital, Suzhou, Jiangsu 215131 China

**Keywords:** High flow nasal cannula, Intravenous anesthesia, Ureteroscopic holmium laser lithotripsy, Elderly, Recovery quality

## Abstract

**Background:**

Intravenous anesthesia with high-flow nasal cannula (HFNC) has been reported to benefit oxygen reserves and enhance postoperative recovery in surgeries requiring low neuromuscular blockade. This study investigated whether HFNC improves recovery quality in elderly undergoing ureteroscopic holmium laser lithotripsy (UHLL).

**Methods:**

We enrolled 106 elderly patients undergoing UHLL, with 96 patients (48 per group) included in the final analysis. Patients were randomly assigned (1:1, stratified by sex) to receive either HFNC (HFNC group) or laryngeal mask airway (LMA) assisted general anesthesia (LMA group). The primary outcome was the Quality of Recovery 15-questionnaire (QoR-15) scores. Secondary outcomes included PACU stay duration, time to out-of-bed mobilization, length of hospital stay, airway dryness scores, surgeons’ satisfaction, and postoperative complications.

**Results:**

Compared to the LMA group, the HFNC group achieved significantly higher QoR-15 scores (125.5 [118.3–130.0] vs. 136.5 [126.3–139.0]; difference = -9, 95%CI, -11 to -5; *P* < 0.001) on the first postoperative day. For secondary outcomes, the HFNC group had a shorter PACU stay (difference = 11.6 min, 95% CI, 10.4–12.8 min), earlier out-of-bed mobilization (difference = 31.8 min, 95% CI, 30.6–33.1 min), lower mouth (difference = 2, 95% CI, 1–3) and throat dryness scores (difference = 2, 95% CI, 1–3) at 30 min post-operation, and lower rates of postoperative sore throat (14.6% vs. 0%; *P* = 0.019) and cough with sputum (odds ratio [OR] = 9.4, 95% CI, 1.1–78.4). No significant differences were observed between the groups for other measures.

**Conclusions:**

HFNC can improve recovery quality in elderly patients after UHLL compared to LMA-assisted general anesthesia.

**Trial registration:**

This trial was registered on July 20, 2023, in the Chinese Clinical Trial Registry (ChiCTR2300073757).

## Introduction

Urolithiasis is the most common indication for urological surgery. Elderly patients, with a generally weakened immune system, face a higher risk of severe infections due to persistent urolithiasis [1]. Delayed treatment can result in urinary tract infection, obstruction, and potentially secondary damage to the kidneys—a particularly dire situation for elderly patients [2]. Ureteroscopic holmium laser lithotripsy (UHLL) is a highly effective treatment for ureteral stones [3]. Currently, general anesthesia with mechanical ventilation is the standard approach for this procedure in elderly patients [4]. However, elderly patients with this method may experiences prolonged recovery from anesthesia and postoperative pulmonary complications [5]. Additionally, endotracheal intubation and laryngeal mask placement can cause damage to the teeth, throat mucosa, or trachea [6–8]. All of these clinically relevant complications can be seriously detrimental to elderly’s comfort, affect the sleep and eating, and even pose a threat to the perioperative rehabilitation [8]. Therefore, it is crucial to develop a potential anesthesia protocol to accelerate elderly’ recovery after urological surgery.

The guidelines from the European Association of Urology (EAU) recommend the use of local anesthesia and intravenous anesthesia, especially in female patients [9]. While UHLL under local anesthesia has been previously explored, it can sometimes induce anxiety, delirium, hypertension, and pain [10, 11]. Intravenous sedation with nasal oxygen can facilitate this procedure, yet risks remain as physiological changes in elderly patients, including loss of static lung compliance, stiffness of the chest wall, and reduced alveolar surface area, may limit oxygen storage and potentially cause hypoxia during surgery [12]. As the current anesthesia management methods are still not optimal, we innovatively combine intravenous anesthesia with High-flow nasal cannula (HFNC) oxygen therapy for this surgery. HFNC provides high-flow oxygen to maintain optimal oxygenation, generates positive airway pressure contributing to lung recruitment, and offers heated and humidified oxygen-enriched air to preserve the integrity of the ciliary mucous system during anesthesia [13–15]. Recently, HFNC oxygen therapy combined with intravenous anesthesia has demonstrated positive outcomes in preventing postoperative respiratory complications in various surgeries with low neuromuscular blockade requirements, including thoracic, laryngeal, and tracheoscopic procedures [16–18]. However, the benefits of intravenous anesthesia with HFNC for elderly patients undergoing UHLL remain unexplored.

The main objective of this randomized controlled trial was to answer the research question: whether intravenous anesthesia with HFNC compared to laryngeal mask airway assisted mechanical ventilation general anesthesia could improve postoperative recovery quality among elderly patients undergoing UHLL.

## Methods

### Patients

Patients aged 60 to 85 years, with an American Society of Anesthesiologists (ASA) physical status of I-III, a Body Mass Index (BMI) between 18 and 30 kg/m² and stones from 4 mm to 15 mm who were undergoing elective UHLL, were recruited for this randomized controlled trial. The exclusion criteria included impacted stones, sepsis, sleep apnea syndrome, asthma, severe respiratory insufficiency, a history of COVID-19 infection, recent myocardial infarction, uncontrolled hypertension, high-risk coronary artery disease, severe hepatic and renal insufficiency, and gastroesophageal reflux.

### Randomization and blinding

Randomization (1:1 ratio, with block sizes of 2 and 4, stratified by sex) was performed using the Sealed Envelope online randomization tool (https://www.sealedenvelope.com/simple-randomiser/v1/lists). The randomization was stratified by gender, considering that female patients tend to experience poorer postoperative recovery. The resulting random allocations were enclosed within sequentially numbered opaque envelopes and sealed before surgery began. After the induction of anesthesia, an independent researcher, who was unaware of the randomization process, unveiled the envelopes and assigned patients to either the HFNC group or the laryngeal mask airway (LMA) group. The anesthesiologists responsible for patient care were informed of the study medications and anesthesia method, while the surgeons and other members of the healthcare team remained uninformed. The subjects, clinicians (excluding the anesthesiologists), and investigators involved in patient recruitment and outcome assessment were completely blinded to the group assignments. The two assessors did not access anesthesia records and were not involved in direct patient care.

### Anesthesia and study interventions

Baseline HR, arterial blood pressure (via radial artery cannulation), 3-lead electrocardiography (ECG), peripheral oxygen saturation (SpO₂), and bispectral index (BIS; Medtronic, Minneapolis, MN, USA) were monitored throughout the procedure. After the subjects were situated in the operating room, a single 10 ml dose of 2% lidocaine was administered and retained in the urethra for 10–15 min.

In the LMA group, general anesthesia was induced using i.v. propofol 1–2 mg/kg and fentanyl citrate 0.5–1.5 µg/kg. Neuromuscular blockade was achieved with cisatracurium benzoate 0.15 mg/kg for laryngeal mask placement. After laryngeal mask placement, the mechanical ventilation was set the IPPV(Intermittent Positive Pressure Ventilation) mode, and the parameters were tidal volume of 6 to 8 ml/kg, appropriate positive end-expiratory pressure, and a respiratory frequency of 12–16 breaths/min. A normal end-tidal carbon dioxide (CO_2_) tension (35 to 45mmHg) was maintained by adjusting the respiratory frequency and the tidal volume intraoperatively. Anesthesia was maintained using remifentanil hydrochloride 0.1–0.3 µg/kg/min and propofol 3–5 mg/kg/h, with adjustments to maintain BIS values between 40 and 60. During surgery, cisatracurium 0.03 mg/kg bolus injections were administered as needed for neuromuscular blockade. After surgery, the patients would get reversal agents according to the anesthetist’s decision.

In the HFNC group, general anesthesia was induced using i.v. fentanyl 0.5–1.5 µg/kg and propofol 1–2 mg/kg. Anesthesia was maintained at the targeted depth (BIS 50–80) by manually adjusting i.v. propofol 1–2 mg/kg/h and remifentanil 0.1–0.3 µg/kg/min. No neuromuscular blockers were used, allowing subjects in the HFNC group to maintain spontaneous breathing. Oxygen was provided via high-flow nasal cannula (HFNC) with humidified oxygen therapy [30.0 L/min; FiO₂, 100%; and gas temperature, 37.0 °C]. If the pulse oxygen level fell below 93%, the attending anesthesiologists increased the oxygen flow to maintain normal oxygenation. Tracheal intubation could be considered if necessary for safety. If movement occurs during the operation, a single 5 ml dose of 2% lidocaine was administered and retained in the urethra for 3 min, meanwhile additional propofol 30–50 mg was used intravenously.

For both groups, dexamethasone (5 mg) and ondansetron (4 mg) were administered to prevent postoperative nausea and vomiting (PONV). Patients experiencing intraoperative blood pressure below 80% of the baseline value were administered intravenous ephedrine (5 mg), phenylephrine (40 µg), or underwent rapid fluid replacement. After surgery, patients were transferred to the Post-Anesthesia Care Unit (PACU), where the level of anesthesia recovery was assessed using the Aldrete score.

### Study outcomes

The primary outcome was the QoR-15 scores after surgery, as defined by the QoR-15 questionnaire [19]. This global assessment tool evaluates postoperative recovery across five dimensions: physical comfort (5 items), physical independence (2 items), emotional state (4 items), psychological support (2 items), and pain (2 items). Each item is rated on an 11-point scale, with higher scores indicating greater frequency of positive outcomes and lower frequency of negative outcomes. The overall score ranges from 0 (indicating the poorest quality of recovery) to 150 (indicating the best quality of recovery).

Secondary outcomes primarily included the length of PACU stay, time to out-of-bed mobilization, airway dryness scores [20], rate of postoperative sore throat, cough, sputum, and surgeons’ satisfaction (three surgeons have consistent professional title level, rich clinical experience and rigorous scientific research attitude). Safety outcomes were assessed, including hypoxemia (defined as a pulse oxygen level < 93% for at least 1 min), hypotension (defined as a reduction in mean arterial pressure (MAP) > 30% of the baseline value for at least 1 min), hypertension (defined as an increase in MAP > 30% of baseline for at least 1 min), bradycardia (defined as heart rate (HR) < 50 beats per minute for at least 1 min), and tachycardia (defined as HR > 100 beats per minute for at least 1 min) during surgery and in the PACU. Sedation in the PACU (defined as an Observer’s Assessment of Alertness/Sedation Scale (OAA/S) ≤ 3) and the occurrence of headache, dizziness, nightmares, or hallucinations within 0–24 h after surgery were also considered. Symptoms (nausea, vomiting, nightmares, hallucinations, and delirium) were documented during ward visits by blinded assessors. Hemodynamic events, interventions, sedation in the PACU, and the duration of PACU stay were recorded in the Surgical Anesthesia Information System (Hangzhou Zejin Information Technology Co., Ltd, Hangzhou, China). Postoperative opioid consumption, rescue analgesia, occurrences of sore throat, cough, sputum, and the length of postoperative hospital stay were documented in electronic medical records and nursing notes. 7-day follow-up data were obtained via telephone. All information was collected in the electronic case report form, which was reviewed by the principal investigator (JL) and an independent data monitoring committee.

### Sample size

Our primary outcome was the quality of recovery (QoR-15) score 24 h post-surgery. The minimum clinically important difference (MCID) in QoR-15 score after surgery is 8 [21], and the standard deviation (SD) of QoR-15 scores (range, 1-150) is typically 10–16. We considered a difference in the mean QoR-15 scores between groups of 8 as clinically significant. We selected an SD of 13 to best reflect our study population. Assuming a two-sided α of 0.05 and a power of 80%, sample sizes were calculated using PASS 15 software, resulting in N1 = 43 for the LMA group and N2 = 43 for the HFNC group. Considering a possible dropout rate of 20%, a minimum of 53 subjects was required for each group, totaling at least 106 study subjects.

### Statistical analysis

The assessment of data distribution was conducted using the Shapiro-Wilk test, and the results are presented as mean (standard deviation [SD]), median (interquartile range [IQR]), or number (%), as appropriate. Perioperative data and study outcomes were compared using the Mann-Whitney U test, chi-square test, or Fisher’s exact test, as deemed appropriate. The estimated effect size was reported in the form of risk difference or relative risk for binary outcomes, hazard ratio for time-to-event, and mean or median differences for continuous data with confidence interval (CI). We defined subjects with BMI > 25 as overweight. We categorized patients who had a QoR-15 score < 121 at 24 h post-surgery as having a moderate-poor quality of recovery. We divided the QoR-15 scores into poorer early postoperative recovery (QoR-15 score < 121) and better early postoperative quality of recovery (QoR-15 ≥ 121) [22]. Furthermore, prespecified subgroup analyses were performed on the primary outcome by sex (female vs. male), current smoking status (no vs. yes), and BMI scores (≤ 25 vs. > 25). For the secondary outcomes, multiple comparisons were not corrected; thus, these results should be interpreted as exploratory. Interim analysis or missing data imputation was not performed. The statistical analysis was performed using SPSS software (version 25.0, IBM SPSS Inc). A two-sided P-value of less than 0.05 was considered indicative of a statistically significant difference.

## Results

### Patient characteristics

**Fig. 1:**
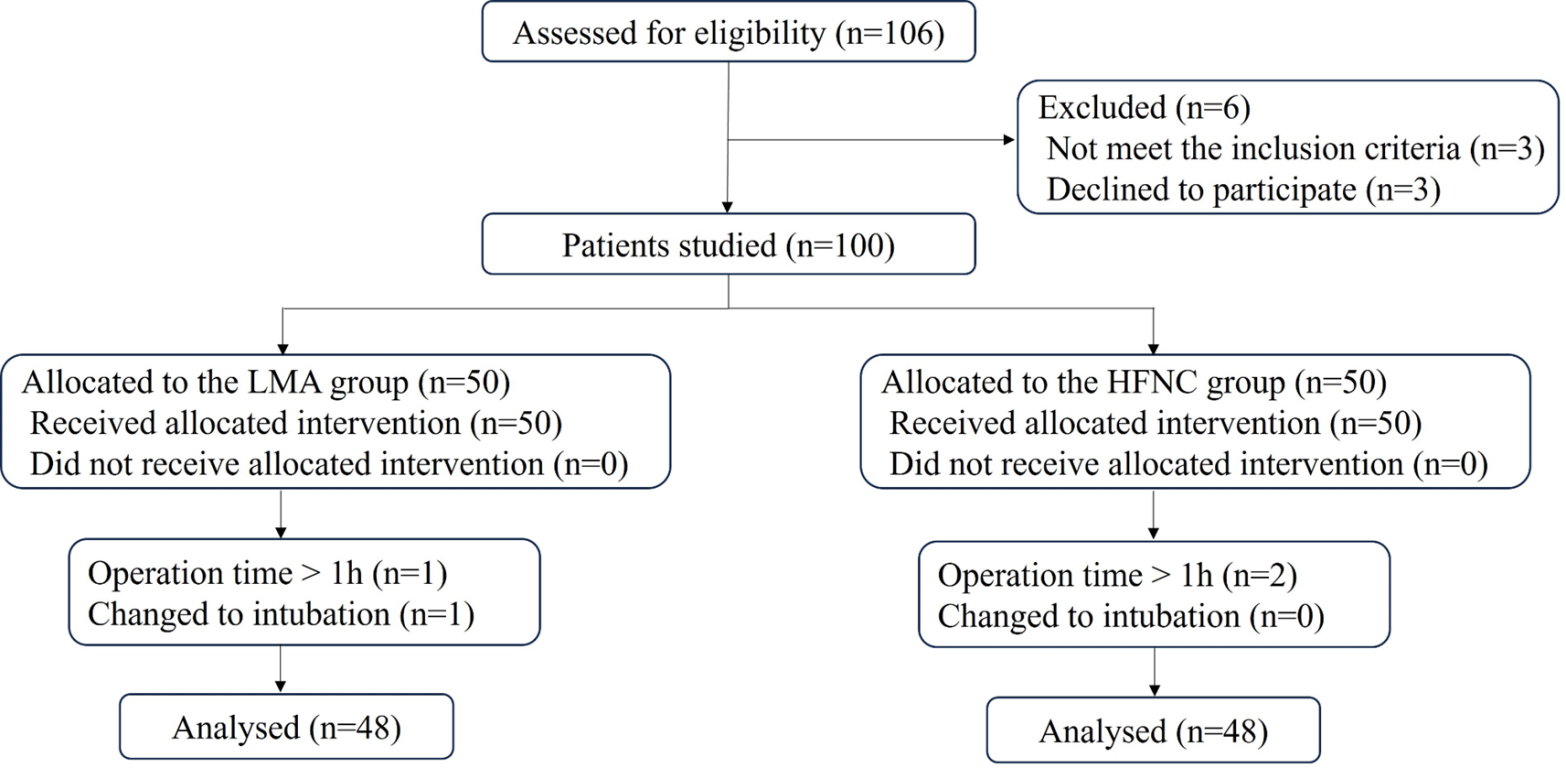
Trial flow diagram. LMA, laryngeal mask airway; HFNC, high flow nasal cannula

From July 2023 to December 2023, a total of 106 patients were screened (Fig. 1). Of these, 6 were excluded, and 100 were randomly assigned to either the HFNC or LMA group. All randomized patients received their assigned anesthesia regimens during surgery. Finally, 96 patients were included in the analysis; 3 were excluded due to operation time exceeding 1 h, and 1 patient required intubation.

The baseline characteristics between the two groups are presented in Table 1. The mean (SD) age was 73 (4) in the HFNC group and 72 (3) in the LMA group. BMI averaged 23.8 (2.3) kg/m² in the HFNC group and 24.4 (2.8) kg/m² in the LMA group. Most patients were classified as ASA physical status II (86.5%). In both groups, 60.4% (58 of 96) of patients were male. The two groups had comparable preoperative baseline cardiorespiratory function. The stone location and size were similar between both groups. Overall, the patient demographics, surgical, anesthetic characteristics, laboratory biochemical indexes and respiratory related index were similar between the groups.

**Table 1:** Patients and baseline characteristics

	LMA (*n* = 48)	HFNC (*n* = 48)	*P*-value
Age, y	72.0(3.6)	73.0(4.0)	0.192
BMI, kg/m2	24.4(2.8)	23.8(2.3)	0.234
ASA status I II III	2(4.2%) 42(87.5%) 4(8.3%)	1(2.1%) 41(85.4%) 6(12.5%)	0.803
Sex male female	29(60.4%) 19(39.6%)	29(60.4%) 19(39.6%)	> 0.99
Smoking	16(33.3%)	15(31.3%)	0.827
Breath-holding test < 30s ≥ 30s	5(10.4%) 43(89.6%)	8(16.7%) 40(83.3%)	0.371
Pulmonary function, L FVC FEV_1_	3.11[2.7–3.4] 0.67[0.6–0.7]	3.06[2.7–3.3] 0.67[0.6–0.7]	0.965 0.437
Cardiovascular comorbidity Hypertension CHF Arrhythmia	20(41.7%) 0 8(16.7%)	18(37.5%) 0 10(20.8%)	0.676 - 0.601
Hemoglobin, g/dL	13.68(1.3)	13.81(1.4)	0.628
Stone location Proximal Mid Distal	3(6.3%) 9(18.8%) 36(75%)	5(10.4%) 8(16.7%) 35(72.9%)	0.883
Stone size ≤ 10 mm > 10 mm	40(83.3%) 8(16.7%)	42(87.5%) 6(12.5%)	0.563
Preoperative QOR-15 score Preoperative pH Preoperative PaO_2_, mmHg Preoperative P/ F, mmHg Preoperative PaCO_2_, mmHg Preoperative RR Preoperative Airway dryness score Mouth Throat	140.5[138.25-143.75] 7.39[7.36–7.42] 76.73(4.84) 365.38(22.93) 39[36–42] 16[15–16] 4[4–5] 4[4-4.8]	141.0[135.25–144.0] 7.36[7.35–7.41] 76.10(5.58) 362.40(26.58) 36[35–41] 16[16–16] 4[4–4] 4[4–4]	0.869 0.125 0.367 0.367 0.097 0.388 0.054 0.218

Data are mean (SD), median [IQR], or n (%)

Abbreviations: SD, standard deviation; IQR, interquartile range; BMI: body mass index; FVC: forced vital capacity; FEV1: forced expiratory volume in 1s; CHF: Chronic heart failure; pH, potential of hydrogen; PaO_2_, partial pressure of oxygen; P/F, PaO_2_ / fraction of inspired oxygen ratio; PaCO_2_, arterial partial pressure of carbon dioxide; RR, respiration rate

**Table 2:** Perioperative data

	LMA (*n* = 48)	HFNC (*n* = 48)	OR or difference (95%CI)	*P*-value
Postoperative pH	7.39[7.36–7.41]	7.38[7.35–7.40]	0.01(0-0.02)	0.137
Postoperative PaO_2_, mmHg	74 (5)	75(5)	1.07(-2.93-1.31)	0.920
Postoperative P/ F, mmHg	353(25)	357(24)	5.08(-13.96- 6.22)	0.920
Postoperative PaCO_2_, mmHg	44[41–47]	45[42–48]	-1(-3-0)	0.116
Postoperative RR	16[16–16]	16[16–16]	0(0–0)	0.894
Body movement	1(2.1%)	3(6.25%)	3.13(0.31–31.25)	0.610
Remifentanil consumption, ug	131[104–158]	74[49–112]	49(32–67)	< 0.001
Fentanyl consumption, ug	50[46.3–60]	55[50–60]	0(-5-0)	0.293
Propofol consumption, mg	205[183–227]	169[134–219]	30(10–47)	0.005
Length of surgery, min	31(10)	32(10)	-1.40(-5.45-2.65)	0.495
stone-free rate	100	100	-	-

Data are mean (SD), median [IQR], or n (%)

Abbreviations: CI, confidence interval; pH, potential of hydrogen; PaO_2_, partial pressure of oxygen; P/F, PaO_2_ / fraction of inspired oxygen ratio; PaCO_2_, arterial partial pressure of carbon dioxide; RR, respiration rate

**Table 3:** Primary outcomes, secondary outcomes and safety outcomes

	LMA (*n* = 48)	HFNC (*n* = 48)	OR or difference (95%CI)	*P*-value
Primary outcomes				
QOR-15 score				
POD1	125.5[118.3–130.0]	136.5[126.3–139]	-9(-11 - -5)	< 0.001
POD2	134.5[123.3-146.5]	141.5[125.8–145]	-1(-7-2)	0.523
POD3	142.5[135–145]	141.5[132–149]	-1(-4-3)	0.577
POD7	142.5[135.3–145]	142[138–149]	-1(-4-1)	0.270
Secondary outcomes				
Duration of PACU stay, min	24.8(3.1)	13.2(2.8)	11.60(10.42–12.79)	< 0.001
Time to first out-of-bed, min	94.9(3.2)	63.1(3.1)	31.81 (30.57–33.05)	< 0.001
Length of hospital stay, d	2[2–2]	2[2–2]	0(0–0)	0.849
Sore throat	7(14.6%)	0(0%)	-	0.019
Cough and phlegm	8(16.7%)	1(2.1%)	9.4(1.13–78.41)	0.036
Airway dryness score				
Mouth				
30 min after surgery	5[4.3-6]	3[2–4]	2(1–3)	< 0.001
1 h after surgery	4[3-5.8]	3.5[2–5]	1(0–1)	0.058
3 h after surgery	2[2–2]	2[1–2]	0(0–0)	0.096
Throat				
30 min after surgery	5[4–7]	3[2–4]	2(1–3)	< 0.001
1 h after surgery	4[3-4.8]	4[3.3-4]	0(0–0)	0.919
3 h after surgery	2[2-2.8]	2[1–2]	0(0–0)	0.109
Surgeons’ satisfaction				0.504
Not satisfied	0	0		
Satisfied	6(12.5%)	4(8.3%)		
Totally satisfied	42(87.5%)	44(91.7%)		
Safety outcomes				
Hypotension	5(10.4%)	4(8.3%)	0.782(0.197–0.859)	> 0.99
Bradycardia	3(6.3%)	4(8.3%)	1.364(0.288–6.448)	> 0.99
Hypertension	1(2.1%)	2(4.2%)	2.043(0.179–23.319)	> 0.99
Tachycardia	2(4.2%)	3(6.3%)	1.533(0.245–9.614)	> 0.99
Interventions for haemodynamic events	9(18.8%)	7(14.6%)	0.740(0.251–2.180)	0.584
Hypoxemia	2(4.2%)	3(6.25%)	1.53(0.45–9.61)	> 0.99
Sedation in PACU	4(8.3%)	2(4.2%)	0.478(0.083–2.744)	0.673
PONV within 0–48 h	3(6.3%)	2(4.2%)	0.652(0.104–4.089)	> 0.99
Nightmare or hallucination	0(0)	0(0)		-
Delirium	0(0)	0(0)		-

Data are mean (SD), median [IQR], or n (%)

Abbreviations: CI, confidence interval; QOR-15, Quality of Recovery 15-questionnaire (QoR-15); PACU, post-anesthesia care unit; PONV, postoperative nausea and vomiting

### Perioperative data

Compared with the LMA group, the HFNC group had lower opioid consumption throughout anesthesia and surgery. In the HFNC group, the median [IQR] dose of remifentanil was 74 [49, 112] µg. In the LMA group, the median [IQR] dose of remifentanil was 131 [104, 158] µg. The mean length of surgery was 31 (10) min in the LMA group and 32 (10) min in the HFNC group. Body movement occurred in three patients in the HFNC group, while one patient in the LMA group experienced body movement. This difference, however, was not statistically significant.

In terms of lung function assessment, the two groups had comparable results, including pH (potential of hydrogen), PaO₂ (partial pressure of oxygen), P/F (oxygenation index), PaCO₂ (partial pressure of carbon dioxide), and RR (respiration rate) during the perioperative period (Table 2).

### Primary outcomes

**Fig. 2:**
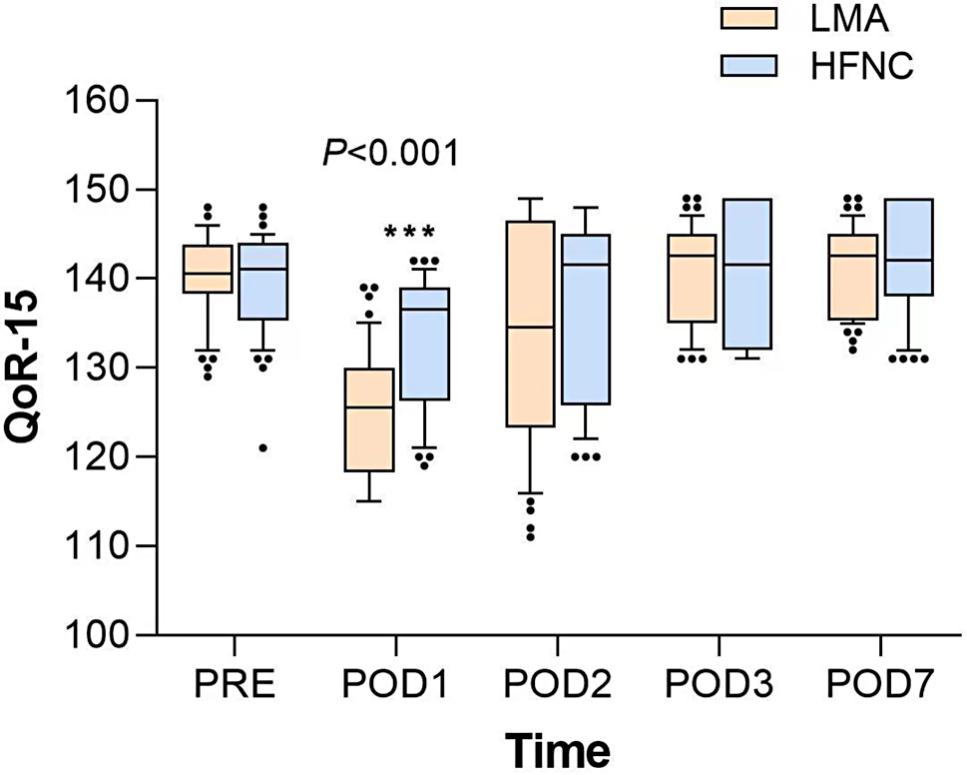
Comparision of the QOR-15 scores of LMA and HFNC group at PRE, POD1, POD2, POD3 and POD7. Data were expressed as median (horizontal bar), interquartile range (box) and the outliers (points). QOR-15, Quality of Recovery 15-questionnaire (QoR-15); LMA, laryngeal mask airway; HFNC, high flow nasal cannula; PRE, pre-operation; POD, post-operation day

The primary outcomes are presented in Table 3; Fig. 2. Compared with the LMA group, the HFNC group had a significantly higher postoperative QoR-15 score (125.5 [118.3–130.0] vs. 136.5 [126.3–139.0]; median difference = -9, 95% confidence interval [CI], -11 to -5; *P* < 0.001) at the first 24 h post-surgery. The differences were not statistically significant on postoperative days 2, 3, and 7.

### Secondary outcomes

The length of PACU stay was longer in the LMA group (mean difference = 11.6 min, 95% CI, 10.4–12.8 min). The time to first out-of-bed mobilization for patients in the HFNC group was significantly shorter than in the LMA group (mean difference = 31.8 min, 95% CI, 30.6–33.1 min). There was no significant difference in the length of postoperative hospital stay or surgeons’ satisfaction between the two groups. Compared with the LMA group, airway dryness scores (mouth and throat) 30 min after surgery were lower in the HFNC group. During the first 24 h after surgery, 7 patients (14.6%) in the LMA group experienced sore throat compared to none in the HFNC group (*P* = 0.019). Cough and sputum occurred in 8 patients (16.7%) in the LMA group vs. 1 patient (2.1%) in the HFNC group (OR = 9.4, 95% CI, 1.1–78.4; *P* = 0.036).

### Safety outcomes

All safety outcomes exhibited no significant differences between the two groups (Table 3). Hypotension occurred in 8.3% of subjects in the HFNC group and 10.4% in the LMA group, with bradycardia occurring infrequently in both groups. Transient hypoxia events during the operation were treated in 6.3% of patients in the HFNC group and 4.2% in the LMA group. Additionally, two patients in the LMA group developed postoperative hypoxia upon removal of the laryngeal mask.

### Stratified analysis

**Fig. 3:**
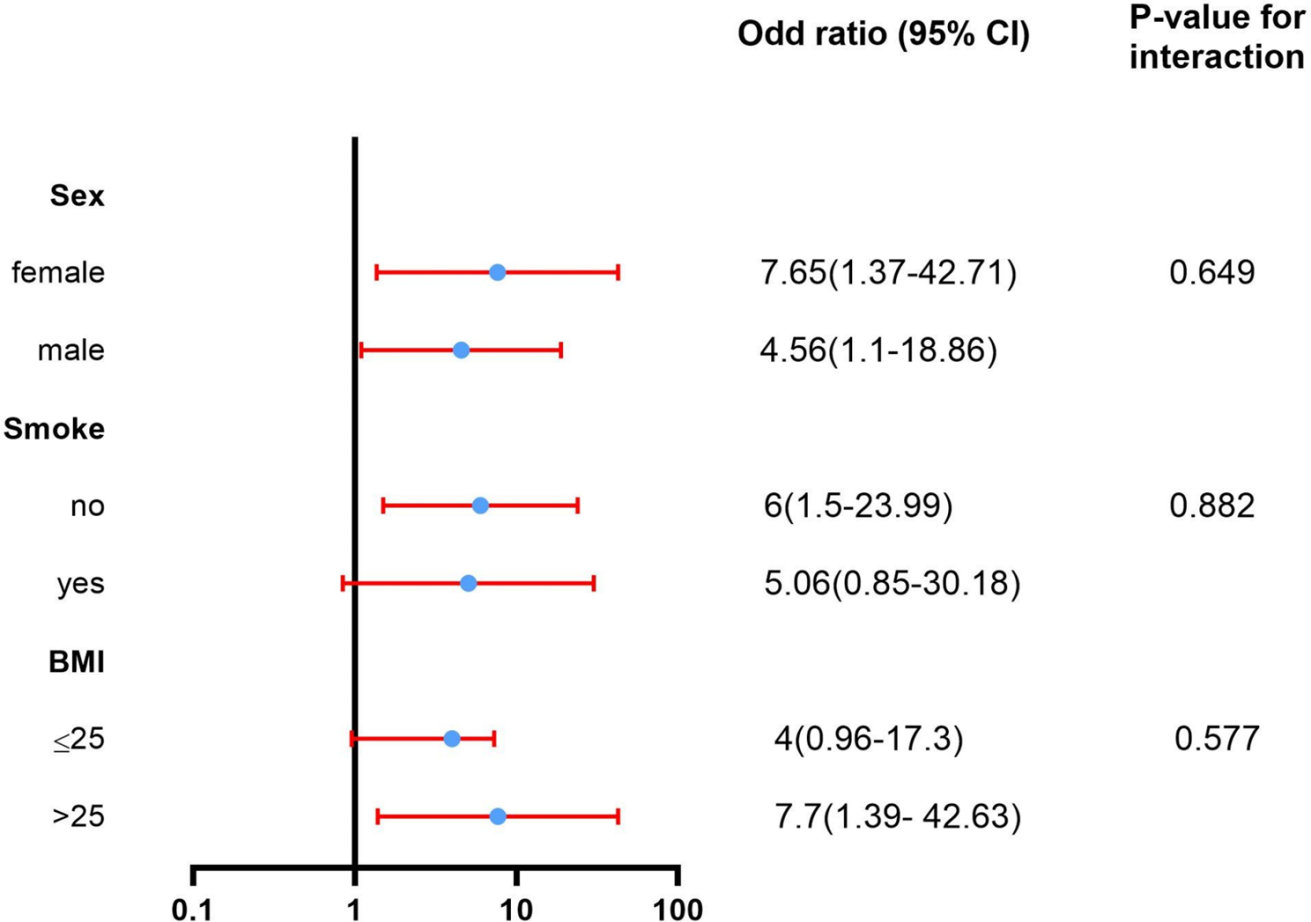
Subgroup analysis of the QOR-15 scores at POD1. BMI: body mass index; CI, confidence interval

In predefined subgroup analyses, the two groups were comparable in terms of treatment effects on postoperative QoR-15 scores within the subgroups of sex (female vs. male, *P* = 0.649), smoking status (no vs. yes, *P* = 0.882), and BMI (≤ 25 vs. >25, *P* = 0.577) (Fig. 3).

## Discussion

In this randomized controlled trial, we demonstrated that monitored anesthesia care with HFNC led to a statistically and clinically significant increase in the quality of postoperative recovery among older patients undergoing UHLL within the first 24 h, compared with laryngeal mask airway anesthesia. Additionally, monitored anesthesia care with HFNC decreased opioid consumption, airway dryness scores, length of PACU stay, and time to out-of-bed mobilization. The safety outcomes were comparable between the two anesthesia regimens.

Over the past few years, HFNC oxygen therapy has been applied in various medical procedures, including anesthesia induction, sedation, gastroenteroscopy, and fiberoptic bronchoscopy [18, 23, 24]. Additionally, a randomized study indicated that HFNC enhances arterial oxygenation during one-lung ventilation in non-intubated thoracoscopic surgery [25]. Transnasal Humidified Rapid-Insufflation Ventilatory Exchange (THRIVE), a form of HFNC, could even keep patients with mild systemic disease and a BMI < 30 well-oxygenated for up to 30 min [26]. These studies suggest that HFNC oxygen therapy is feasible and safe for surgery, potentially providing optimal oxygenation levels, positive airway pressure, and maintaining the integrity of the ciliary mucous system [27, 28]. Moreover, another study showed that HFNC had the potential to reduce postoperative recovery time, decrease the occurrence of agitation, and enhance lung function and oxygenation status during the anesthesia recovery period [29]. In our study, 48 elderly patients received monitored anesthesia care with HFNC; all successfully completed the operation, and the success rate of lithotripsy was 100%.

A BIS level between 40 and 60 is typically considered the ideal anesthesia state. Monitored anesthesia care with HFNC provides smoother airways compared to laryngeal mask-assisted mechanical ventilation general anesthesia. The risk of laryngospasm is much lower with HFNC compared to the use of a LMA. Therefore, in our study, we implemented a novel strategy of maintaining a higher BIS range of 50–80 within the HFNC group. Research suggests that maintaining BIS in the range of 61–70 and 71–80 can lead to reduced propofol usage during surgery, better quality of recovery among elderly patients, and decreased incidents of hypotension [30]. Based on experiences with ureteroscopy lithotripsy using 100 µg fentanyl induction under sedation and anesthesia [31], elderly patients require appropriate dosage adjustments. For fentanyl use, the median [IQR] dose in the HFNC and LMA groups was 55 [50,60] and 50 [46.25,60] µg, respectively. We opted to combine remifentanil with propofol for anesthesia maintenance. This pairing offers fast action, rapid metabolism, easy control, and optimal safety, and decreases postoperative fatigue, thereby promoting faster recovery. The primary outcome in our trial used QoR-15, which demonstrated strong efficacy, reliability, and sensitivity. Chazapis et al. reported that most patients’ QoR-15 scores had returned to baseline levels by 48 h postoperatively and exceeded pre-surgery levels by seven days [32]. Hence, focusing on QoR-15 scores obtained before surgery or within the first seven days post-surgery yields more accurate results. Our trial findings provide compelling clinical evidence that monitored anesthesia care with HFNC notably elevates the quality of recovery within 24 h postoperatively, compared with the LMA group.

The secondary outcomes also indicated that monitored anesthesia care with HFNC could enhance recovery after surgery in older patients undergoing UHLL within the first 24 h. A certain level of neuromuscular blockade is often needed when using LMA during general anesthesia. However, insufficient recovery from neuromuscular blocking drugs is linked to adverse outcomes, such as upper airway obstruction, reintubation, atelectasis, pneumonia, prolonged stay in the PACU, and reduced patient satisfaction [33]. Additionally, the increased use of opioids during anesthesia might adversely impact the immune function of older patients, potentially detracting from the quality of early recovery [34]. The findings in Table 3 indicate a notable decrease in airway dryness scores, postoperative sore throat, and postoperative cough and sputum in the HFNC group. Moreover, patients in this group demonstrated improved communication with medical staff, relatives, and friends during the early postoperative period. These positive changes enhanced the quality of early recovery in three QoR-15 clusters: psychological and emotional state, pain, and physiological adaptability. The length of PACU stay and time to out-of-bed mobilization were both reduced in the HFNC group, which is essential for the expulsion of residual crushed stones from the urine after UHLL [35].

The QoR-15 has strong construct validity, revealing a negative correlation with both duration of surgery and total opioid use [36]. Thus, we standardized the duration of surgery in this study to minimize bias between the groups. In line with reported studies, our results showed that monitored anesthesia care with HFNC could decrease total opioid use, enhancing recovery after surgery. In addition, previous evidence indicates that women experience worse postoperative recovery. (37–38) To avoid gender bias, we performed a prespecified stratified analysis, which showed that the treatment effects of the LMA group versus the HFNC group on QoR-15 scores did not differ in the subgroups of sex.

This study has several limitations. Firstly, it was conducted at a single hospital raising questions about its generalizability beyond this specific setting, and further protocol will be performed for multi-center trial. Secondly, only ASA I-III patients with a BMI below 30 were included, limiting the applicability of the findings to patients with advanced cardiopulmonary disease or a higher BMI. Thirdly, only the LMA group received neuromuscular blocking drugs. The better outcome in the HFNC group could be due to the patients not having received any neuromuscular blocking agents. Although reversal agents may be used in the study protocol according to the needs of the anesthesiologist, there is still the possibility of neuromuscular blocking agents residues. In the future, LMA group can be treated without neuromuscular blocking agents, which could rule out this as a potential confounding factor. Lastly, we did not perform measurements for hypercapnia by transcutaneous carbon dioxide in real time during the surgery, as this study mainly focused on the postoperative recovery. However, previous research has shown that HFNC applied to spontaneously breathing patients can increase CO_2_ clearance and decrease the respiratory rate due to dead space wash-out [39].

In conclusion, monitored anesthesia care with HFNC can improve the postoperative recovery quality of older patients undergoing UHLL and decrease opioid use, airway dryness scores, length of PACU stay, and time to out-of-bed mobilization. This regimen is feasible, safe, and beneficial for UHLL.

## Data Availability

The datasets used in this research may be made available upon reasonable request to the corresponding author.
